# Computational model of integrin adhesion elongation under an actin fiber

**DOI:** 10.1371/journal.pcbi.1011237

**Published:** 2023-07-06

**Authors:** Samuel Campbell, Michelle C. Mendoza, Aravind Rammohan, Matthew E. McKenzie, Tamara C. Bidone

**Affiliations:** 1 Department of Biomedical Engineering, University of Utah, Salt Lake City, Utah, United States of America; 2 Department of Oncological Sciences, University of Utah, Salt Lake City, Utah, United States of America; 3 Huntsman Cancer Institute, University of Utah, Salt Lake City, Utah, United States of America; 4 Corning Life Sciences, Tewksburry, Massachusetts, United States of America; 5 Corning Research and Development Corporation, Corning, New York, United States of America; 6 Scientific Computing and Imaging Institute, University of Utah, Salt Lake City, Utah, United States of America; National Institutes of Health, UNITED STATES

## Abstract

Cells create physical connections with the extracellular environment through adhesions. Nascent adhesions form at the leading edge of migrating cells and either undergo cycles of disassembly and reassembly, or elongate and stabilize at the end of actin fibers. How adhesions assemble has been addressed in several studies, but the exact role of actin fibers in the elongation and stabilization of nascent adhesions remains largely elusive. To address this question, here we extended our computational model of adhesion assembly by incorporating an actin fiber that locally promotes integrin activation. The model revealed that an actin fiber promotes adhesion stabilization and elongation. Actomyosin contractility from the fiber also promotes adhesion stabilization and elongation, by strengthening integrin-ligand interactions, but only up to a force threshold. Above this force threshold, most integrin-ligand bonds fail, and the adhesion disassembles. In the absence of contraction, actin fibers still support adhesions stabilization. Collectively, our results provide a picture in which myosin activity is dispensable for adhesion stabilization and elongation under an actin fiber, offering a framework for interpreting several previous experimental observations.

## Author summary

The formation of cell adhesions is important for numerous biological processes, including cell migration, differentiation, survival, tissue morphogenesis, and wound healing. However, to fully understand how adhesions control biological processes, we need to unravel the biophysical principles underlying their assembly and maintenance. Experimentally, assessing how adhesion proteins form and maintain adhesions is difficult. The mechanisms by which adhesion proteins regulate adhesions are not well understood, and controversy exists on the roll of cell contractility. Here, we developed a model of adhesion assembly and characterized how adhesions elongate in the presence and absence of contraction. We found that force is not needed for the transition of adhesions into elongated morphologies.

## Introduction

The ability of cells to form adhesions with the extracellular environment is critical to many physiological and pathological processes, including wound healing, tissue morphogenesis, embryonic development, and cancer metastasis [1–3]. Adhesions are hierarchical assemblies of ~ 150 proteins that associate sequentially on the cell membrane, starting from transmembrane integrin receptors undergoing activation and connecting extracellular matrix (ECM) ligands to the actin cytoskeleton [4–6]. During cell spreading and migration, nascent adhesions form in a thin region at the cell protruding edge, called the lamellipodium. In the lamellipodium, actin filaments promote inside-out integrin activation and sustain edge motion [7–11]. Nascent adhesions either rapidly turn over or stabilize to the ECM near the base of the lamellipodium, where the actin filaments change their architecture from branched to bundled and form radial fibers, under which the adhesions elongate. Nascent adhesions under the actin fibers can further grow into focal complexes and mature adhesions through the recruitment of intracellular accessory proteins, such as talin, vinculin, paxillin, and zyxin, among others [12–17]. It is well established that the maturation of integrin-based adhesions involves the sequential recruitment of several accessory proteins, actomyosin contractility and intracellular signaling [18–21], but how nascent adhesions elongate at the end of actin fibers remains largely elusive.

Nascent adhesions appear in the lamellipodium as clusters containing an average of 20–50 integrins, with a diameter of ~100 nm, smaller than the ~0.25 *μ*m of the light microscope [4, 22]. They typically disassemble within seconds or minutes [4]. When they do not disassemble, they transition into elongated morphologies and connect with actin filaments in stress fibers. In these adhesions, individual integrins are highly dynamic and undergo cycles of ligand binding and unbinding, governed by inside-out activation through talin binding to the integrin cytoplasmic tails [11, 22–24].

Actomyosin contractility from the actin fibers stabilizes nascent adhesions and supports integrin connections with the ECM through reinforcement of their bonds with ECM ligands. Experiments with myosin inhibitors and dominant negative mutants have shown that actomyosin force also promotes the recruitment of intracellular accessory proteins and the maturation of adhesions [15, 25–28]. However, several experiments have demonstrated that adhesion elongation does not require myosin activity [29, 30], which suggests that a force-independent stabilization of the nascent adhesions may precede myosin-mediated reinforcement. Without myosin activity, substrate stiffness is needed to support the stabilization of adhesions [31]. However, how exactly adhesions elongate and stabilize under an actin fiber is unknown.

**Fig 1:**
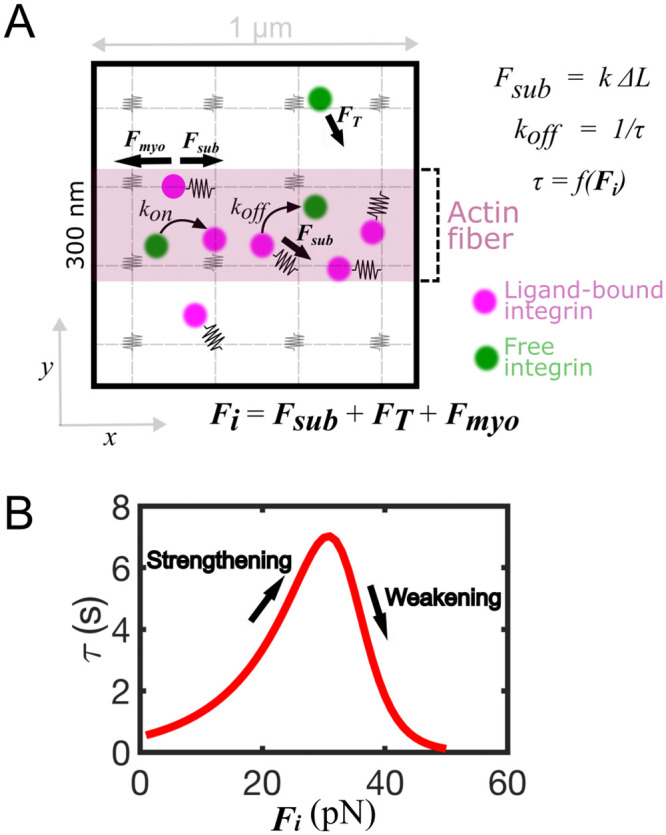
Computational model of integrin-based adhesion assembly in the presence of an actin bundle. **A**. Schematics of the 2D computational model. The domain consists of a grid of ideal springs (gray particles) with stiffness *k*. Integrins diffuse (green particles) with diffusion coefficient *D* and establish harmonic interactions with the substrate springs (magenta particles). If integrins are in the region of the actin fiber (pink area) a force, ***F***_***myo***_ is also added. ***F***_***myo***_ build tension on the integrin-ligand bond. This tension determines the bond lifetime, *τ*, which is the reciprocal of the rupture rate, *k*_*off*_, as *τ* = *k*_*off*_. **B.** The bond lifetime versus force relation for integrin-ligand bonds follows catch-slip bond kinetics.

In this study, we sought to understand the mechanisms of adhesion elongation and stabilization at the end of an actin fiber by testing the contributions of actomyosin force, inside-out activation, and substrate stiffness to these mechanisms. Because the transient nature of nascent adhesions makes it difficult to distinguish adhesion assembly from elongation and stabilization, here we applied computational modeling (a schematic of the model is provided in Fig 1).

Our results revealed that an increase in the fiber contractile activity alone stabilizes the adhesion up to a certain force threshold. Above this force threshold, most integrin-ligand bonds fail, and the adhesion disassembles. In the absence of contraction, inside-out activation of integrins under an actin fiber accounts for adhesion stabilization and elongation. Taken together, the results from our model demonstrate a myosin-independent mechanism for adhesion elongation, which is regulated by inside-out integrin activation.

## Results

### Overview of the computational model

We extended our computational model of adhesion assembly based on overdamped Langevin dynamics [29, 32] by incorporating the effect of an actin fiber. Our simulation domain was simplified to a 2D square of 1 μm side (Fig 1A). Ligands were fixed at the vertices of a 20x20 lattice. Integrins were represented as point particles that diffuse and transition between inactive to active states, with defined probabilities. When they were active and in proximity of a free ligand, integrins could establish harmonic interaction potentials with that ligand and become ligated. The duration of each bond was determined by the probability of rupture, *k*_*off*_, which was calculated from the total force on the bond. In particular, the lifetime (*τ* = 1/*k*_*off*_) of the integrin-ligand bonds followed catch-slip kinetics, in which an initial strengthening of the bond occurred with increasing force, followed by weakening at higher forces (Fig 1B). The effect of an actin fiber was incorporated in the model by considering a stripe of 300 nm width, at the center of the domain (Fig 1A). The fiber locally changed the kinetic of integrins underneath it. Under the fiber, integrin activation was increased, depending on the degree of bundling of actin filaments, and a force (actomyosin force in the fiber, ***F***_***myo***_) was applied to all ligated integrins. Depending on the spring constant of the harmonic interaction between integrin and ligand, which mimicked substrate stiffness and contributed to the substrate force, ***F***_***sub***_, and depending upon the magnitude of ***F***_***myo***_ in the fiber region, the total force on the integrin-ligand bonds was calculated and the probability of rupture determined. Activation, ligand binding and unbinding resulted in cycles of integrin diffusion, activation, and de-activation, which mimicked nascent adhesions. The main objective of the model was to test the effect of an actin fiber on the orientation and stability of the adhesion. The effects that fiber contractility had on several adhesion properties were evaluated by running simulations in the presence and absence of contraction, and using varying ***F***_***myo***_. The effect of substrate stiffness, which further contributed to the total force on the integrin-ligand bonds, was also assessed.

### Integrin adhesions are elongated under an actin fiber

**Fig 2:**
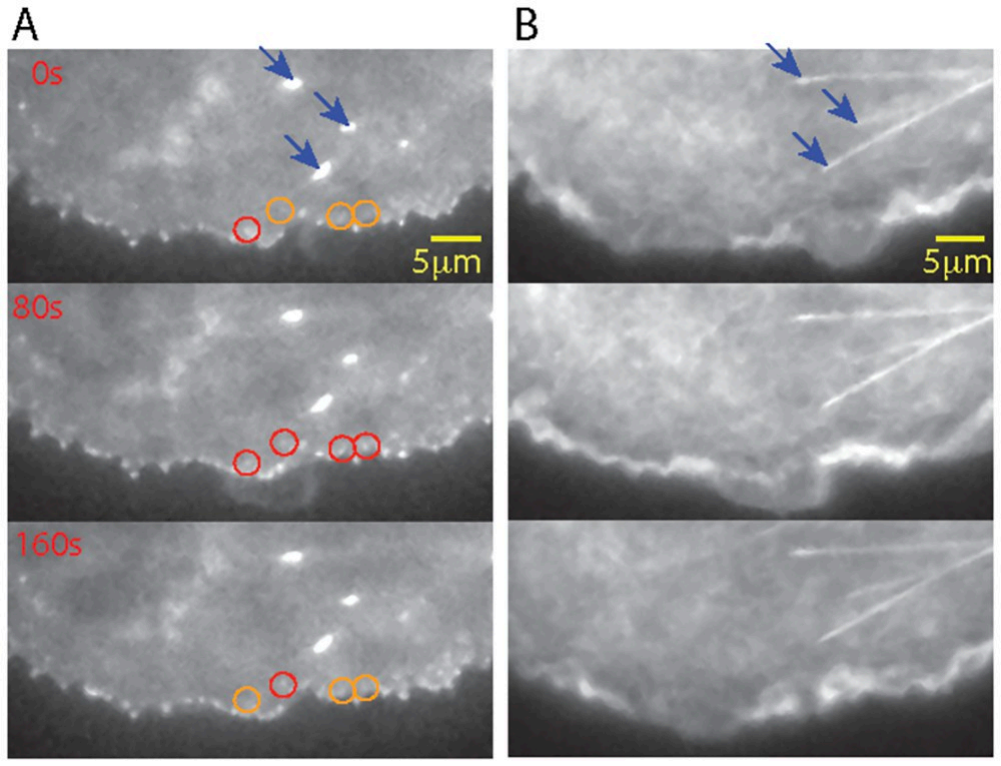
Experimental image showing adhesion elongation and stabilization under actin fibers. COS7 cell, transiently transfected to co-express mScarlet-paxillin and EGFP-F-tractin. **A.** mScarlet-paxillin imaged by Total Internal Reflection Fluorescence Microscopy (TIRFM) and **B.** EGFP-F-tractin imaged by epifluorescence. Edge motion is shown in 80s intervals. Nascent adhesions (circles) assemble (orange to red) and disassemble (red to orange) as the leading edge moves forward. Stabilized adhesions under bundled actin fibers (blue arrows) do not disassemble.

We confirmed that integrin adhesions are elongated under actin fibers using COS7 kidney epithelial cells. Cells were co-transfected with mScarlet-paxillin to label adhesions and EGFP-F-tractin to label actin. Imaging by TIRF showed adhesions assembling at the advancing leading edge (Fig 2A), and larger elongated adhesions were found only back from the edge and under a thick actin fiber (Fig 2A and 2B). These data suggest that actin fibers have a role in the elongation of integrin adhesions at the back of the leading edge, consistent with previous reports [2, 33, 34].

### Actomyosin contractility and substrate stiffness interplay in the assembly of adhesions

**Fig 3:**
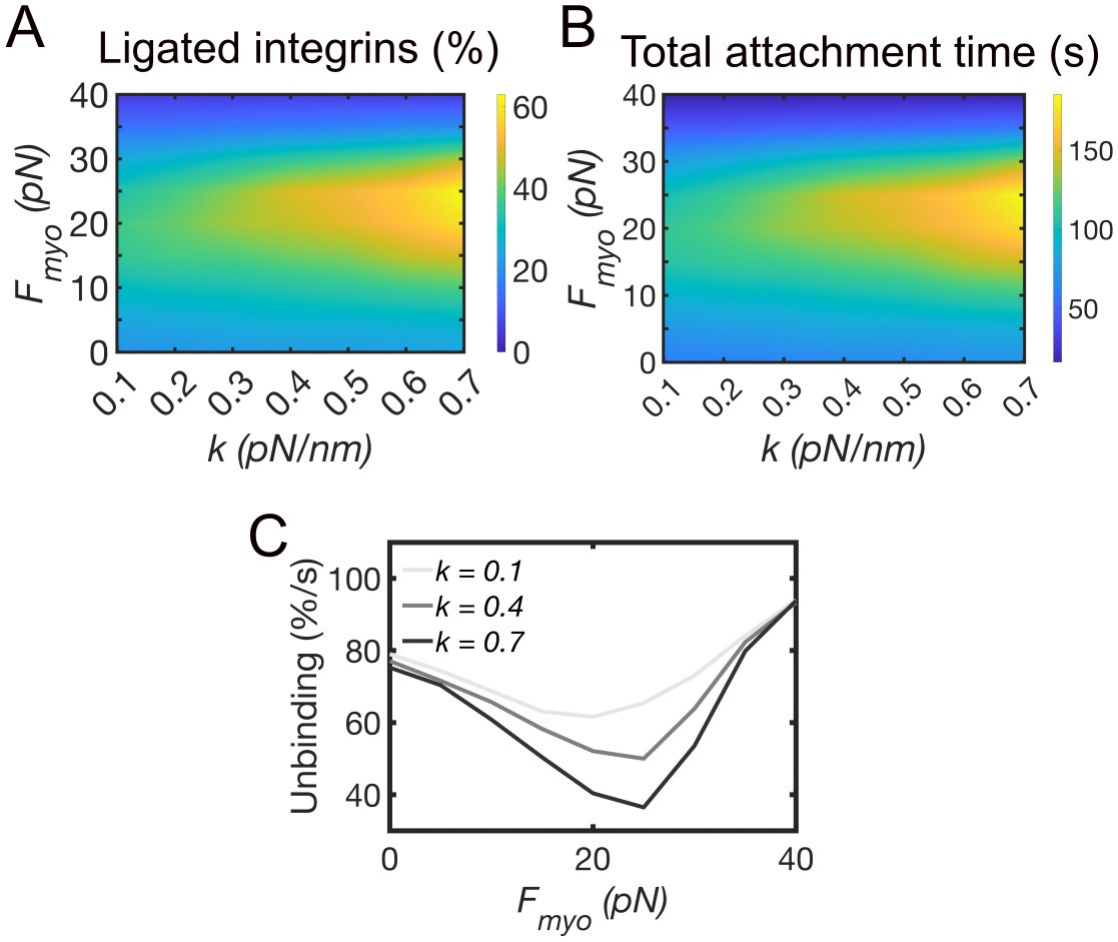
Actomyosin force combined with substrate stiffness result in load-and-fail of adhesions. **A.** A heatmap of the average percentage of ligand bound integrins varying substrate stiffness, *k*, between 0.1–0.7 pN/nm, and actomyosin force, ***F***_***myo***_, between 0–40 pN. The reported values are averages between 100–300 s of simulations. **B.** A heatmap of the total ligated time of integrins during 300 s of simulations, varying *k* between 0.1–0.7 pN/nm and ***F***_***myo***_ between 0–40 pN. **C.** The average percentage of integrins undergoing unbinding every second of simulations, varying ***F***_***myo***_ between 0–40 pN, using *k* = 0.1, 0.4, and 0.7 pN/nm. Data are extracted between 100–300 s of simulations.

Since actomyosin force and ECM stiffness play an important role in the maturation of integrin adhesions [18, 34–36], we tested how actomyosin force from fibers can account for the stabilization of nascent adhesions. We ran simulations by systematically varying the magnitude of actomyosin force, ***F***_***myo***_, up to 40 pN, and substrate stiffness, *k*, from 0.1 to 0.7 pN/nm. The average percentage of integrin-ligand bonds first increased with increasing ***F***_***myo***_, then it decreased, and this effect was more marked at high substrate rigidities (Fig 3A). For ***F***_***myo***_ = 15–30 pN, an increase in ligated integrins was observed with increasing *k*, which corresponded to an increase of the total time spent by integrins in the ligated state (Fig 3B). A maximum of ~ 55–65% of ligated integrins was reached for ***F***_***myo***_ = 15–30 pN and *k =* 0.6–0.7 pN/nm (Fig 3A). A percentage of 55–65% corresponded to a minimum separation between ligated integrins of ~70 nm, corresponding to the maximum interligand distance for adhesion stabilization [37, 38], thus indicating a high probability of adhesion stabilization. For ***F***_***myo***_ > 30 pN, the percentage of ligated integrins dropped below 20% at all substrate rigidities, indicating low probability of adhesion stabilization. Because the activation rate of integrins was fixed, while their unbinding rate depended on the force on the integrin-ligand bonds (Fig 1B), the amount of unbinding was different for different values of ***F***_***myo***_ and *k*. For ***F***_***myo***_ = 15–30 pN, the rate of unbinding decreased with increasing substrate stiffness from *k* = 0.1 pN/nm to *k* = 0.7 pN/nm (Fig 3C). The reduced unbinding of integrins resulted from the strengthening phase of the catch-slip bond, which emerged from the combined effect of actomyosin contractility and substrate stiffness (Fig 1B). For ***F***_***myo***_ > 30 pN, unbinding increased on all substrates, because of the weakening phase of the catch-slip bond (Fig 1B).

Together, these results indicated that actomyosin contractility and substrate stiffness need to be finely tuned to stabilize adhesions by reducing integrin unbinding. Increasing actomyosin contractility up to 30 pN, which is the force corresponding to the maximum lifetime of the catch-slip bond (Fig 1B), maximally reduces the unbinding of integrins. Increasing substrate stiffness augments this effect. When actomyosin contractility is higher than 30 pN the integrin-ligand bonds fail (Fig 3A and 3C), and the adhesion is unstable on all substrates.

### Actin fibers supports elongation of adhesions

**Fig 4:**
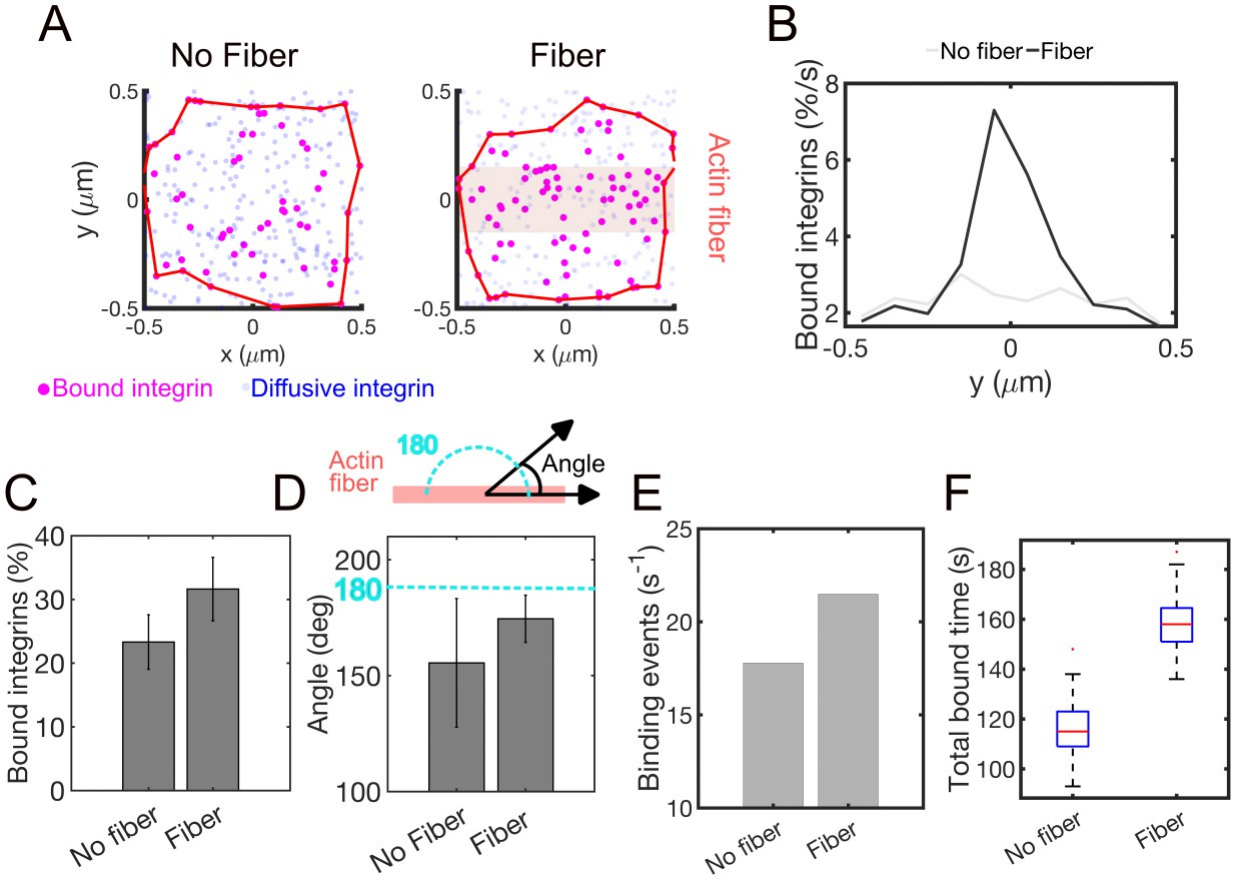
Bundling of actin filaments stabilizes the assembly of nascent adhesions. **A.** Snapshots of ligand-bound integrins (magenta) at 200 s of simulations, using no fiber (left) and a fiber (right; the pink rectangle indicates the location where the actin fiber overlaps with the adhesion). **B.** The average percentage of ligated integrins along the fiber short axis in the absence and presence of the fiber. **C**. Average percentage of ligated integrins using *P*_*bundling*_ = 0 (no fiber) and *P*_*bundling*_ = 1 (fiber). Errorbars indicate standard deviation from the mean. **D**. Average angle of the adhesion, relative to the direction of the actin fiber, using *P*_*bundling*_ = 0 (no fiber) and *P*_*bundling*_ = 1 (fiber). The angle is calculated from the direction of the first principal component of the 2D positions of ligated integrins, computed at each second of simulations between 100–500 s. Errorbars indicate standard deviation from the mean. **E.** Frequency of binding events using *P*_*bundling*_ = 0 (no fiber) and *P*_*bundling*_ = 1 (fiber). **F.** Distribution of total time spent by integrins in the ligated state, calculated as sum of the ligand-bound lifetimes of for each integrin over the course of 500 s of simulations, using *P*_*bundling*_ = 0 (no fiber) and *P*_*bundling*_ = 1 (fiber). All data are computed in the absence of actomyosin contractility, from 3 independent runs using *k* = 0.6 pN/nm.

Since results from our model showed that actomyosin contractility first lead to adhesion stabilization, but then lead to adhesion failure (Fig 3A and 3C), we tested the hypothesis that actin filaments in a fiber, by promoting inside-out integrin activation, stabilize nascent adhesions. By comparing simulations without and with an actin fiber, we found that ligated integrins concentrate under the fiber (Fig 4A and 4B). The higher density of ligated integrins under the fiber was reflected in a higher total percentage of ligated integrins (Fig 4C). Like the case without an actin fiber (Fig 3A), incorporation of actomyosin contractility further increased the percentage of ligated integrins up to a threshold force above which bond failure occurred and the percentage of ligated integrins decreased (S1A Fig). Bond failure was due to the higher force on the integrin-ligand bonds resulting from application of the actomyosin force (S1B Fig). The actin fiber oriented the adhesion along the axis of the fiber (Fig 4D). Incorporation of actomyosin contractility and increasing the width of the actin fiber further increased the alignment of the adhesion with the fiber (S1C Fig). The total time that integrins spent in the ligated state presented a biphasic response to ***F***_***myo***_, because of the two phases of the catch-slip bond. This occurred both without and with an actin fiber, implying that only the force on the integrin-ligand bond modulates this effect and not the fiber (S2A and S2B Fig). Using an actin fiber in the absence of contraction, the rate of ligand binding increased from ~18 s^-1^ to about ~23 s^-1^ (Fig 4E), which increased the total time spent by integrins in the ligated state by ~ 50% (Fig 4F). Increasing the width of the actin fiber increased the percentage of ligated integrins in the adhesion (S3A Fig), the number of binding events per second (S3B Fig), and the total time that integrins spend in the ligated state (S3C Fig). However, by increasing the width of the fiber, the adhesion first aligned with the fiber; then, above a fiber width of 300 nm, this alignment decreased (S3D Fig). The differences in adhesion orientation with fiber width suggest that the elongation of integrin adhesions originates from a local rather than global effect.

Taken together, our results show that an actin fiber, by promoting integrin activation and without contraction, can increase integrin binding of substrate ligands, enhance the lifetime of integrin-ligand bonds, and promote the elongation of the adhesion along the fiber. These results can explain how adhesions change at the base of the lamellipodium, where the actin filaments change architecture from branched to bundled and contractility is not significant.

### Interplay between actin fiber contractility and bundling on adhesion stabilization

**Fig 5:**
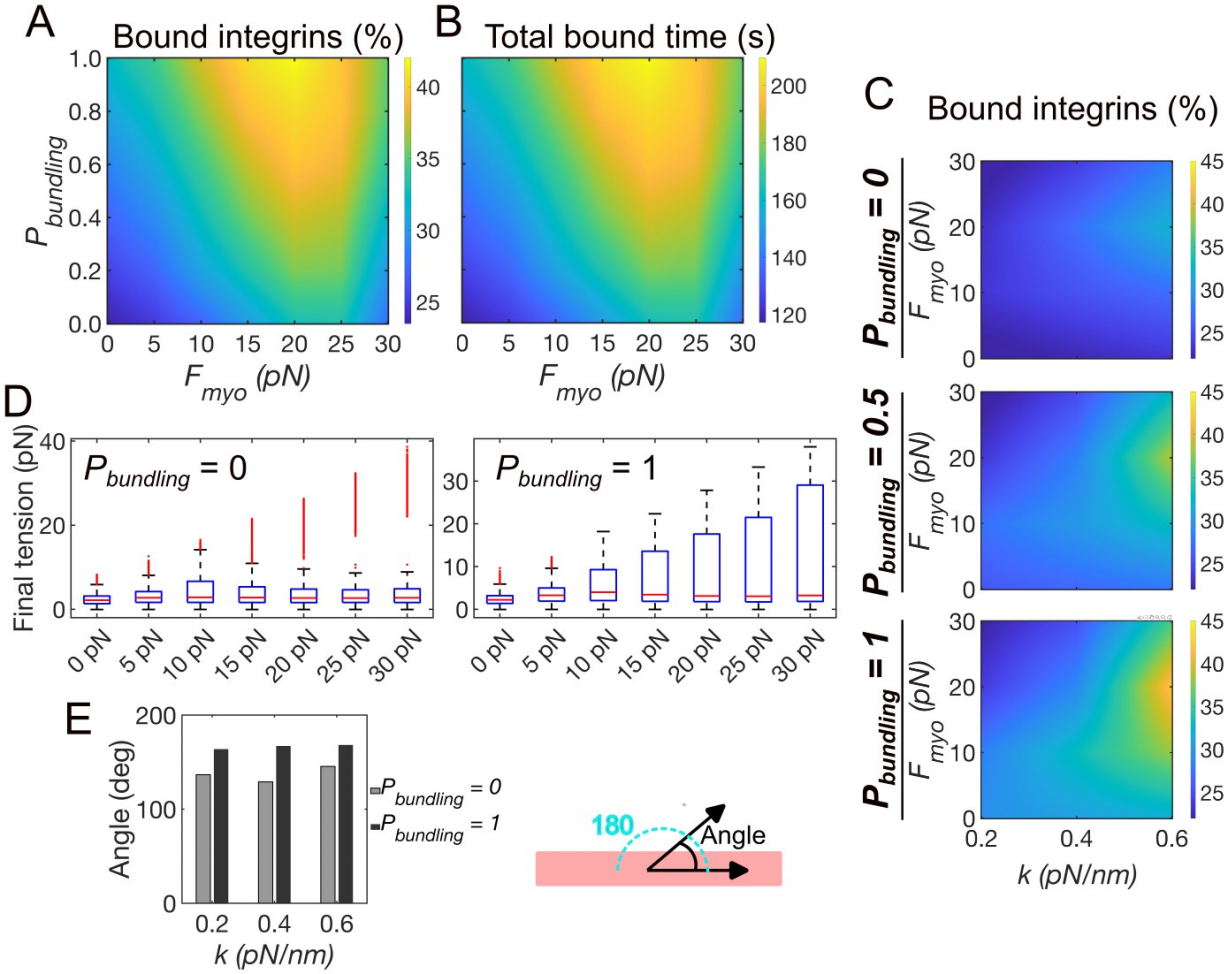
With high actin bundling, actomyosin tension has no effects on adhesion stabilization. **A.** The average fraction of ligand-bound integrins varying *P*_*bundling*_ between 0–1, and ***F***_***myo***_ between 0–30 pN and using *k* = 0.6 pN/nm. **B.** The average total ligand-bound time for integrins, varying *P*_*bundling*_ between 0–1, and ***F***_***myo***_ between 0–30 pN and using *k* = 0.6 pN/nm. Data are computed as averages from three independent simulations. **C.** The average fraction of ligand-bound integrins for probabilities of actin filaments bundling *P*_*bundling*_ = 0, 0.5, and 1, varying ***F***_***myo***_ between 0–30 pN and *k* between 0.2–0.6 pN/nm. **D.** Distribution of the final tension on the integrin-ligand bonds before failure varying ***F***_***myo***_ between 0–30 pN, using *P*_*bundling*_ = 0 and 1, and fixed substrate stiffness at *k* = 0.6 pN/nm. All data are computed between 100–300 s of simulations, from three independent runs. **F.** The average angle of integrin adhesions relative to the fiber axis using *P*_*bundling*_ = 0 and 1, using ***F***_***myo***_ = 0 and *k* between 0.2–0.6 pN/nm. The angle is calculated from the direction of the first principal component considering the 2D positions of ligated integrins, at each second of simulation.

The results from our model showed that an actin fiber can support the elongation and stabilization of an integrin adhesion in the absence of contraction (Fig 4). Since actin fibers are typically contractile and present varying numbers of crosslinking proteins and motors [39–41], we examined how contractility and actin bundling affects adhesions. We assessed how systematic variations in the probabilities of actin bundling, *P*_*bundling*_, and actomyosin contractility, *F*_*myo*_, affect the density of ligand-bound integrins, the time that integrins spent in the ligated state, and the orientation of the adhesion. By increasing *P*_*bundling*_, the average percentage of ligated integrins increased from ~25%, up to ~ 50%, depending on *F*_*myo*_ (Fig 5A). Without an actin fiber (*P*_*bundling*_ = 0), a four-fold increase in ***F***_***myo***_, from 5 pN to 20 pN, increased the average percentage of ligated integrins from ~25% to ~35%, a total increase of about 40% (Fig 5A). Using low actomyosin contractility, ***F***_***myo***_ = 5 pN, increasing *P*_*bundling*_ from 0 to 1 increased the average percentage of ligand-bound integrins of the same amount (Fig 5A). With ***F***_***myo***_ = 20 pN, varying *P*_*bundling*_ from 0 to 1 increased the fraction of ligand-bound integrins from ~35% to ~45%, corresponding to a total increase of ~30%. However, with higher actomyosin force, ***F***_***myo***_ = 30 pN, the same increase in *P*_*bundling*_ increased the fraction of ligated integrin from ~30% to only ~35%, a total increase of ~16%. These results indicated that, like the effect of contractility, actin filaments bundling in the fiber also supports adhesion stabilization. When acting together, bundling augment the effect of contractility to stabilize adhesions. However, when actomyosin contractility is high, the effect from bundling on adhesion stabilization is reduced. Therefore, actin bundling alone or with intermediate contractility maximally supports adhesion stabilization. The observed increases in the fractions of ligated integrins as a function of *P*_*bundling*_ originated from the increased activation rate of integrins under the fiber, resulting in a longer time that integrins spent in the ligated state (Fig 5B). For ***F***_***myo***_ = 5 pN, increasing *P*_*bundling*_ from 0 to 1 increased the total bound time of ~40% (Fig 5B), an amount comparable to the corresponding variation in the percentage of ligand-bound integrins under the same conditions (Fig 5A). For ***F***_***myo***_ = 20 pN, the same increase in *P*_*bundling*_ increased the total time spent by integrins in the bound state of ~15% (Fig 5B), again comparable to the increase in percentage of ligated integrins under the same conditions (Fig 5A). Increasing substrate stiffness together with ***F***_***myo***_ stabilized the adhesion at all levels of *P*_*bundling*_ (Fig 5C), with the more marked effect occurring when *P*_*bundling*_ = 1. At *k* = 0.6–0.7 pN/nm and ***F***_***myo***_ = 20 pN, the maximum value of ligated integrins increased from ~35% at *P*_*bundling*_ = 0, to ~ 40% using *P*_*bundling*_ = 0.5, and ~ 45% using *P*_*bundling*_ = 1 (Fig 5C). Plots of the distribution of the tension before bond breakage showed comparable average values between absence and presence of bundling but increases in the spread of the distribution with increasing ***F***_***myo***_ using *P*_*bundling*_ = 1 (Fig 5D). Actin bundling also affected the orientation of the adhesion, by promoting its alignment along the fiber on all substrates (Fig 5E).

Collectively, results from our model demonstrate that an actin fiber supports adhesion elongation on all substrates and augment the effect of contractility in stabilizing adhesions.

## Discussion

Nascent adhesions form in the lamellipodium of adherent cells and elongate at the end of actin fibers radiating perpendicularly to the cell edge [4]. Experiments probing the mechanisms of adhesion elongation and stabilization at the end of actin fibers have variably suggested a requirement for contractile force [3, 28, 42–44] versus bundling of the actin filaments in the fiber [4, 45]. As a result, the mechanisms governing elongation and stabilization of adhesions are not well established. In this study, we extended our computational model of integrin adhesion assembly based on Brownian dynamics [29, 32] to test the contributions of actomyosin contractility and actin bundling to the dynamics of adhesions. Results from this study revealed that an actin fiber, by providing a physical template for inside-out integrin activation, guides the elongation of nascent adhesions in the direction of the fiber (Figs 4 and 5). Forces from actomyosin contractility also promote adhesion stabilization and elongation, but only up to a certain force level, then they lead to adhesion failure and disassembly (Fig 3A and 3B).

Brownian dynamics simulation approaches are widely used for understanding the dynamics of biological macromolecules. Our computational model used this method to reproduce cycles of free diffusion and immobilization of integrins to the ECM. Together with the thermal effects on integrin motion, that governed integrin diffusion, our model also evaluated kinetic rate constants for ligand binding and unbinding, which determined transitions between free and ligated integrins. Displacements of integrins were calculated over time, based on the sum of forces acting on them. When integrins were bound to substate ligands, they were subjected to the force from actomyosin contractility in the fiber region, and the substrate resisting deformation. Force from the substrate varied proportionally with substrate stiffness, *k*, and modulated the rate of bond rupture, *k*_*off*_. *k*_*off*_ decreased with increasing *k*, so that integrins unbound less easily when *k* was high, resulting in an increased average fraction of ligated integrins on stiff substrates (Fig 3). Bundling was incorporated by increasing the rate of integrin activation under the fiber. Variations in substrate stiffness were incorporated using different spring constants for integrin-ligand bonds, as in previous models [46–48]. Thus, the force on the *i*-th ligated integrin, ***F***_***i***_, was the sum of: (*i*) stochastic force from thermal effects, ***F***_***T***_; (*ii*) actomyosin contractility in the region of the actin fiber, ***F***_***myo***_ = 5–40 pN, a range comparable to the contractility of actomyosin bundles in cells [49, 50]; and (*iii*) force from the substrate limiting integrin motion away from the ligand, ***F***_***sub***_, proportional to the substrate stiffness *k* (between 0.1–0.7 pN/nm [46, 51]). Considering only the effects of thermal force on integrin motion, the total force on ligated integrins, ***F***_***i***_, was in the single pN range, comparable to the average force/integrin reported for single talin molecules [52]. In the presence of actomyosin contractility in the fiber region, the force on ligated integrins reached values of few tens of pN, consistent with experimental measurements of maximal force per integrin, up to ~40 pN [53, 54].

Like our previous study of nascent adhesion assembly and sensing of substrate stiffness [29], results from our model showed that substrate stiffness can account for adhesion stabilization (Fig 3A and 3B). With increasing *F*_*myo*_ between 5–40 pN, adhesions initially stabilized, reaching an optimum at *F*_*myo*_ = 25 pN, but then destabilized for *F*_*myo*_ > 30 pN (Fig 3A and 3B). As the load on integrins increased, the lifetime of the integrin-ligand bonds first increased, and then decreased (Fig 1B), following catch-slip bond kinetics [55, 56]. When individual ligand-bound integrins were subjected to forces above 30 pN, the decrease in bond lifetime led to bond failure. Soft substrates did not support adhesions, resulting in reduction of the density of ligated integrins (Fig 3A). Previous studies have shown that cell contraction is not needed for the assembly of adhesions [29]. However, previous studies have also shown that in the absence of an external force, such as substrate stiffness, actomyosin force promotes adhesion stabilization [31]. This result is consistent with our model result that fiber contractility promotes adhesion stabilization on softer substrates (Fig 3).

Our model also demonstrated a critical role for actin bundling in adhesion elongation and stabilization (Fig 4). Actin bundling increased integrin immobilization under the fiber (Fig 4A and 4B), the average density of ligated integrins in the adhesion (Fig 4C) and the alignment of the adhesion with the fiber (Fig 4D). Alignment of the adhesion with the fiber also correlated with the width of the fiber (S1C Fig). The increase in the percentage of ligated integrins from actin bundling resulted in a reduction of their spatial separation, a prerequisite for adhesion stabilization [37, 38]. Actomyosin force and substrate stiffness affected the rupture rate of integrins through modulation of their lifetimes (Fig 1B). We have previously shown that increasing the concentrations of motors and crosslinking proteins in an actin fiber increases the fiber force, but without crosslinkers high forces cannot be sustained, despite the crosslinking activity of the motors [41]. In cells, force generation from motors and motor crosslinking overlap. However, several previous studies implicated myosin crosslinking rather than contractility as important in adhesion elongation [4, 45], which supports our model results that bundling in the absence of contractility stabilize the adhesion (Fig 4). In CHO.K1 ovary epithelial-like cells, a myosin mutant that retained actin cross-linking activity but lacked motor activity rescued adhesion elongation in *myosin IIA*-null cells [4]. Similarly, in U2OS human osteosarcoma cells, actin bundling was sufficient to rescue the inhibition of myosin activity to drive adhesion elongation and increase the lifetime [30, 34, 45]. Results from our model measured the lifetime and the total bound time of individual integrin-ligand bonds and not the lifetime of the whole adhesion. However, with increasing lifetime of individual ligand-bonds, the adhesion lifetime also increases. Therefore, our results are qualitatively consistent with these previous experiments and collectively support a picture in which actin bundling can alone support adhesion elongation in the absence of contraction (Fig 4).

The impact of actin filaments on nascent adhesions from this model also provides a new framework to understand actin-mediated nucleation of nascent adhesions in the lamellipodium. Previous studies have indicated that actin polymerization drives clusters of activated integrins probing for sites of adhesion nucleation [57]. Similarly, Arp2/3-mediated actin branching around integrin sites has been shown to have an important role in the formation of nascent adhesions [58–60]. Our model suggests a mechanism by which actin-mediated inside-out activation of integrins increases the density of ligated integrins under the fiber, and therefore co-localization of filaments and integrins promote adhesion assembly and stability.

Our model assumes that ligated integrins are connected to the actin cytoskeleton through talin [61]. Talin was considered implicitly by applying actin flow and *F*_*myo*_ to ligated integrins. Because the cytoskeletal force was directly transmitted to the integrin-ligand bonds, this force was not buffered by talin unfolding, and vinculin binding to talin was not incorporated. Extensions of this model to incorporate these effects will be considered in future studies. In the current implementation, our model demonstrates that nascent adhesions can elongate in the direction of an actin fiber without talin unfolding, force redistribution and vinculin binding. This supports the idea that, provided that an actin fiber works as a physical template for integrin activation, talin unfolding and vinculin recruitment are dispensable for adhesion elongation. This result is consistent with experimental studies showing that: initial cell spreading only requires integrin-ligand bonds and that nascent adhesions elongate in the absence of vinculin [4, 58].

The results from our model are consistent with a myosin-independent mechanism for adhesion elongation. We suggest that the initial phases of elongation of integrin adhesions emerge passively from the actin fiber acting as a template for integrin activation in the absence of other reinforcement mechanisms. It is plausible that the recruitment of mechano-sensitive intracellular proteins stabilizes integrin-ligand bonds on stiff substrates.

Since contractile myosin activity correlates with the maturation of elongated adhesions in the lamella [20, 25, 62] and has been presumed to be the important mechanism for adhesion stabilization [28, 34, 63], it remains plausible that myosin- dependent contractility stabilizes nascent adhesions into focal adhesions and recruits’ proteins for specialized non-lamellipodium functions. Contractility may be involved in adhesion maturation into large adhesions in the cell middle or adhesions with different compositions in scenarios specific to substrate signaling or mechanical stretch.

## Materials and methods

To elucidate how nascent adhesions elongate under actin fibers, we extended our Brownian dynamics model of integrin adhesions assembly [29, 32]. An actin fiber was incorporated implicitly in the model, as a region of space where actin filaments bundling and actomyosin contractility affect integrin dynamics. We used the model to evaluate how systematic variations in the probability of actin filaments bundling and in the magnitude of the actomyosin force affect the percentage of ligand-bound integrins, the total time that integrins remain ligated and the orientation of the adhesion.

### Software availability

The code is available at https://github.com/tamarabidone/adhesions_under_a_fiber.git.

### Simulation domain

The simulation domain is a 2D square of 1 μm side, which corresponds to an area of cell membrane where integrins bind ligands and form an adhesion (Fig 1A). The actin fiber corresponds to a central strip of the domain of 300 nm width, in which bundling and actomyosin force control integrin dynamics. Integrins are initially randomly distributed in the domain and undergo free diffusion. Periodic boundary conditions are used to avoid finite boundary effects on integrin motion. Ligands are represented as fixed nodes on a lattice of 20x20 cells (each cell has sides of 50 nm). Interactions between integrins and ligands are governed by kinetic rates and an adhesion is considered formed when multiple bonds between integrins and ligands exist in the domain.

### Integrin and ligand representations

The model considers 300 integrins and 441 immobilized ligands. Each *i*-th integrin and *j*-th ligand are defined by a 2D position vectors, ***r***_***i***_ and ***r***_***j***_, respectively. The vector ***r***_***i***_ presents *x*, and *y* coordinates of the *i*-th integrin; the vector ***r***_***j***_ presented *x*, and *y* coordinates of the *j*-th ligand. At every timestep of the simulations, components *x* and *y* of ***r***_***i***_ are updated to track integrin displacement, while components *x*, and *y* of ***r***_***j***_ remain fixed.

### Brownian Dynamics simulations via Langevin equation

Recognizing that inertia is negligible on the length and time scales of integrin motion in the plasma membrane, the displacement of each *i-*th integrin depends on the total force on it, ***F***_***i***_, and is governed by the Langevin equation of motion in the limit of high friction [64]:

$$dr_{i}=\frac{F_{i}}{\zeta_{i}}dt$$ (1)

where ***r***_***i***_ is the position vector; *ζ*_*i*_ is the friction coefficient equal to 0.071 pN s/μm, which corresponds to a diffusion coefficient *D* = 0.058 μm^2^/s [23], obtained from Einstein relation as $\zeta_{i}=\frac{k_{B}T}{D}$, where *k*_*B*_*T* = 4.11 pN nm.

The explicit Euler integration scheme is used to displace integrins depending on ***F***_***i***_, *ζ*_*i*_ and *dt*, as:

$$r_{i}\left(t+dt\right)=r_{i}\left(t\right)+\frac{dr_{i}}{dt}dt=r_{i}\left(t\right)+\frac{F_{i}}{\zeta_{i}}dt$$ (2)

### Forces on integrins

The total force on each *i*-th integrin, ***F***_***i***_, includes a stochastic contribution from thermal effects, ***F***_***T***_, and a deterministic contribution from: substrate stiffness, ***F***_***sub***_, and, in the fiber region, actomyosin contractility, ***F***_***myo***_. It is calculated as:

$$F_{i}=F_{T}+F_{sub}+F_{myo}$$ (3)

Thermal force, substrate force, and actomyosin contractility are all calculated at each timestep of the simulations and used to update integrin positions (Eqs 1 and 2). When integrins are diffusing, only ***F***_***T***_ acts on them, while ***F***_***sub***_ = 0 and ***F***_***myo***_ = 0. When integrins are ligated, ***F***_***sub***_ is added. When integrins are ligated and under the actin fiber, ***F***_***myo***_ is also considered.

Since actin flow was shown to govern a rearward movement of integrins in the adhesion, we tested the effects of actin flow velocity on the distribution of force on integrin-ligand bonds. We imposed a displacement of ligated integrins corresponding to experimental actin flows, up to 100 nm/s [8, 65]. Our results showed that the distribution of force on ligated integrins was not affect by actin flow (S4 Fig). Therefore, our model does not include the contribution from the actin flow to the motion of integrins.

### Stochastic force acting on integrins

A stochastic force, ***F***_***T***_, satisfying the fluctuation-dissipation theorem, is applied to all integrins, to mimic thermal effects generating diffusion. ***F***_***T***_ has two force components, where each component is chosen from a Gaussian distribution with average 0, and standard deviation $\sqrt{\frac{2k_{B}T\zeta_{i}}{dt}}$. At each timestep, the 2D displacement of the *i*-th integrin due to the thermal force is computed from the two force components, ***F***_***T*,*x***_ and ***F***_***T*,*y***_, as: $\Delta x_{i}=\frac{F_{T,x}}{\zeta_{i}}dt$; $\Delta y_{i}=\frac{F_{T,y}}{\zeta_{i}}dt$. Therefore, at each *dt* the maximum displacement from thermal effects is ~ 3.5 nm.

### Deterministic forces acting on ligated integrins: Actomyosin force and substrate force

A deterministic force is applied on each ligated *i*-th integrin. It originates from actomyosin contractility in the fiber region, ***F***_***myo***_, and substrate tension, ***F***_***sub***_. ***F***_***myo***_ pushes integrins away from their ligand, in the horizontal direction (Fig 1A). ***F***_***sub***_ counters ***F***_***myo***_ by pulling ligated integrins towards the ligands, to restore the equilibrium distance, *L*.

***F***_***myo***_ mimics contractile motors in the fiber and is applied on ligated integrins in the region where the fiber overlaps with the adhesion. We systematically varied ***F***_***myo***_ between 5–40 pN, which is a range typical of contractile actin bundles [49, 50]. Without considering the opposing substrate stiffness, application of this force corresponds to a displacement $\Delta r_{i}=\frac{F_{myo}}{\zeta_{i}}dt$, in the range 7–42 nm every *dt*.

The force from the substrate, ***F***_***sub***_, is proportional to its stiffness, *k*, and follows Hookes’ law, as:

$$F_{sub}=k\Delta L$$ (4)

where Δ*L* is the deviation from *L*, and *k* = 0.1–0.7 pN/nm, as in previous models [46–48]. Considering *x*_*i*_ and *y*_*i*_ as coordinates of the *i*-integrin and *x*_*j*_ and *y*_*j*_ as coordinates of the bound *j*-th ligand, their separation relative to the equilibrium distance, *L*, is:

$$\Delta L=\sqrt{{\left(x_{i}-x_{j}\right)}^{2}+{\left(y_{i}-y_{j}\right)}^{2}}-L$$ (5)

The displacements of ligand-bound integrin in each direction are calculated as: $\Delta x_{i}=F_{sub}\frac{x_{i}-x_{j}}{\Delta L*\zeta_{i}}dt$; $\Delta y_{i}=F_{sub}\frac{y_{i}-y_{j}}{\Delta L*\zeta_{i}}dt$.

### Binding and unbinding of ligands

Integrins undergo cycles of free diffusion, binding, and unbinding of ligands. When a free, diffusive integrin comes in proximity of a free ligand (< 20 nm from it, which is a dimension characteristic of integrin headpiece extension [66]), it can bind the ligand by establishing an elastic interaction with probability: $p_{on}=1-e^{-k_{on}dt}$, where *k*_*on*_ = 1 s^-1^ is the activation rate of integrin, of the same order of those used in [32], and *dt* = 0.0001 s is the timestep.

The bond between integrin and the ligand has spring constant, *k*, proportional to the substrate stiffness, and equilibrium distance *L* = 0.01 nm. The integrin-ligand bond lifetime, *τ*, depends on the total force on integrin, ***F***_***i***_, and is the inverse of the rupture rate: *τ* = 1/*k*_*off*_. The rupture rate follows a catch-slip bond kinetics, in which the lifetime of the bond initially increases with force ***F***_***i***_, then decreases, as shown in Fig 1B [55]. Following the Bell model [67], *k*_*off*_, is computed from a double exponential function, including a strengthening pathway represented by a first exponential term with a negative exponent; and weakening pathway, represented by a second exponential term with a positive exponent [68, 69], as:

$$k_{off}=2\;e^{-0.1*F_{sub}}+0.000004e^{0.3*F_{sub}}$$ (6)

Since the force on each bond is unique, depending on whether integrin is in the fiber region, and the magnitude and direction of ***F***_***T***_, a specific *τ* exists for each bond. The rupture rate, *k*_*off*_, determines the probability of unbinding as: $p_{off}=1-e^{-k_{off}dt}$. Once integrins unbind their ligands, they become diffusive again until they bind a new ligand.

### Actin fiber representation

In the model, the location where the actin fiber overlaps with the adhesion is incorporated as a 300 nm wide strip in the middle of the domain (Fig 1A). In this location, integrin activation is increased, to mimic cytoplasmic signals promoting integrin conformational extension and an increase in ligand binding affinity, consistent with experimental observations [9, 10].

Actin bundling is incorporated in the code through its effect on integrin activation under the fiber. The probability of actin bundling, *P*_*bundling*_, is the probability that free integrins in the fiber area activate faster. Outside the fiber, integrins activate at a rate *k*_*on*_ = 1 s^-1^, as in our previous study [32]. When the probability of actin bundling is 0.5, about 50% of free integrins under the fiber present *k*_*on*_ = 3 s^-1^ and the remaining 50% present *k*_*on*_ = 1 s^-1^. When the probability of actin bundling is 1, all integrins under the fiber present *k*_*on*_ = 3 s^-1^. The increase in rate of integrin activation under the fiber mimics talin-mediated activation of integrin [70]. Because experimental studies have also indicated that integrins under actin fibers undergo slow and directed movement along the fiber [57, 71, 72], we tested the effect of lowering the diffusion coefficient of free integrins in the fiber region, by increasing *ζ*_*i*_. We found that reducing diffusion increases adhesion alignment in the direction of the fiber, but to a smaller extent than increasing the activation rate (S2C Fig).

### Algorithm implementation

The total simulation time is between 300–500 s, which is a relevant time scale for nascent adhesions assembly and elongation [4]. Results are extracted after 100 s of simulations, when the number of ligated integrins reaches a steady value.

The algorithm consists of two parts: an initialization function and a step function. The initialization function sets the domain geometry and the boundary conditions (periodic in *x* and *y*) and assigns all simulation parameters to specific variables, including integrin diffusion constant, integrin and ligand concentrations and substrate stiffness; then, it sets the initial random positions of integrins and the positions of the ligands in the lattice. The step function runs iteratively, and each iteration corresponds to one timestep, until the total time of 300–500 s is reached. The main objective of the step function is to evaluate the total force on each integrin and update its position and bound state.

### Initialization function

Parameter initialization includes the definition of domain geometry and size, number of integrins (300), and number of ligands (441). Integrins are initially distributed on the 1 x 1 μm domain, by assigning *x* and *y* coordinates randomly between -0.5 and 0.5 μm in both directions. The square domain is further divided into 20x20 cells, for a total of 400 cells, with ligands fixed at their vertices. Since the lateral domain size is 1 μm and there are 20 cells per side, neighboring ligands are separated by 50 nm, a value below the maximum ligand separation of 70 nm needed for adhesion stabilization [37, 38].

### Step function

The step function is divided into three parts: it first evaluates integrin positions; it computes the total force, ***F***_***i***_, acting on each integrin; based on ***F***_***i***_, it updates integrin positions. Transitions between unbound and bound states of integrin are determined by kinetic rates.

The timestep of the simulations is chosen to ensure stability. Decreasing it from *dt* = 0.0001 s to *dt* = 0.00001 s or *dt* = 0.000001 s does not change the results, both in the presence and absence of an actin fiber, and using substates with different rigidities (S5 Fig).

### Cell imaging

COS7 cells were plated for 48 hr prior to imaging in 35 mm Matek (Ashland, MA) glass bottom dishes, at 15,000 cells per dish in Gibco DMEM (Waltham, MA) with 5% FBS (VWR Scientific). Cells were co-transfected with 500 ng pcDNA3.1-mScarlet-paxillin and 300 ng pEGFP-F-tractin (Addgene—Watertown, MA) complexed in Gibco OptiMEM with Mirus TransIT LT1 (Madison, WI) following the manufacturer’s recommended DNA:transfection reagent ratio. Two-channel time-lapse image series were acquired at 2 s intervals (per channel) using a Nikon 100x/1.49NA CFI Apo objective, Nikon TiE Inverted Microscope with Perfect Focus System 3 and motorized TIRF, and a Photometrics Prime 95B camera. mScarlet-paxillin was imaged with 561 nm laser excitation with a 620/60 nm emission filter. EGFP-F-tractin was imaged with 488 nm laser excitation and a 535/45 nm emission filter. Image processing was performed with Fiji software.

## Supporting information

S1 Fig Actomyosin contractility and bundling modulates the orientation of the adhesion relative to the fiber. **A.** Distribution of the percentage of ligated integrins in different conditions of bundling and ***F***_***myo***_ (between 0–30 pN). **B.** Distribution of final tension on ligated integrins in different conditions of bundling and ***F***_***myo***_ (between 0–30 pN). All data are computed between 100–500 s of simulations, extracting values every 1 s. **C.** Average angle of the adhesion using ***F***_***myo***_ = 10 pN (black), 20 pN (dark gray), 30 pN (medium gray), 40 pN (light gray) and two fiber widths: 0.2 and 0.8 nm. Errorbars indicate standard deviation from the mean. The angle is calculated from the direction of the first principal component considering the 2D positions of ligated integrins. All data are computed between 100–500 s of simulations, using *k* = 0.6 pN/nm, and extracting values every 1 s.
(TIFF)

S2 Fig Actomyosin contractility and bundling modulate the total ligated integrins. **A-B.** Distribution of the total time spent by integrins in the ligated state, in different conditions of ***F***_***myo***_ (between 0–30 pN), without (**A**) and with (**B**) actin bundling. **C.** Average angle of the adhesion relative to the actin fiber. Three conditions are tested: absence of bundling; bundling with reduced diffusion of integrin in the fiber region (using 5-fold higher *ζ*_*i*_ than outside the fiber); and bundling with increased activation rate (using 3-fold higher *k*_*on*_ relative to *k*_*on*_ outside the fiber). The angle is calculated from the direction of the first principal component considering the 2D positions of ligated integrins. All data are computed between 100–500 s of simulations, using *k* = 0.6 pN/nm, and recording every 1 s.
(TIFF)

S3 Fig Effect of fiber width on adhesion stability. **A**. Average percentage of ligated integrins varying the width of the actin fiber and using *P*_*bundling*_ = 1. Errorbars indicate standard deviation from the mean. **B.** Number of binding events per second varying the width of the actin fiber and using *P*_*bundling*_ = 1. **C.** Distribution of total time in the ligated state, calculated as sum of the ligand-bound lifetimes of for each integrin over the course of 500 s of simulations, using *P*_*bundling*_ = 1 and increasing the width of the fiber. **D**. Average angle of integrin adhesion, relative to the direction of the actin fiber, using *P*_*bundling*_ = 1 and varying the width of the fiber. The angle is calculated from the direction of the first principal component of the 2D positions of ligated integrins. Errorbars indicate standard deviation from the mean. All data are computed in the absence of actomyosin contractility and are evaluated between 100–500 s of simulations, from 3 independent runs using *k* = 0.6 pN/nm and considering the total number of ligated integrins every 1 s of simulations.
(TIFF)

S4 Fig Effect of actin flow velocity on the distribution of force on the integrin-ligand bonds. **A.** Distribution of force on ligated integrins using actin flow velocity of 0, 10, 30, and 100 nm/s, in different conditions of ***F***_***myo***_ (between 0–30 pN) and using ***F***_***T***_ = 0. **B.** Distribution of force on ligated integrins using actin flow velocity of 0, 10, 30, and 100 nm/s, in different conditions of ***F***_***myo***_ (between 0–30 pN) and including thermal fluctuations. All data are computed between 100–500 s, from 3 independent runs using *k* = 0.6 pN/nm. The distributions are normalized by their maximum value in each plot.
(TIFF)

S5 Fig Effect of the timestep of the simulations on the percentage of ligated integrins. **A.** Average percentage of ligated integrins varying timestep, in the presence (fiber) or absence (no fiber) of actin bundling. When present, a fiber of 300 nm width is used. **B.** Average percentage of ligated integrins varying timestep, and using *k* = 0.2 pN/nm and *k* = 0.4 pN/nm. An actomyosin force of 20 pN is applied to ligated integrins in the fiber area, considering a fiber width of 300 nm. All data are computed between 100–500 s, from 3 independent runs. Errorbars indicate standard error from the mean.
(TIFF)

## References

[1] Hamidi H, Ivaska J. Every step of the way: integrins in cancer progression and metastasis. Nat Rev Cancer. 2018;18(9):533–48. doi: 10.1038/s41568-018-0038-z . 30002479 PMC6629548

[2] Gardel ML, Schneider IC, Aratyn-Schaus Y, Waterman CM. Mechanical integration of actin and adhesion dynamics in cell migration. Annu Rev Cell Dev Biol. 2010;26:315–33. doi: 10.1146/annurev.cellbio.011209.122036 . 19575647 PMC4437624

[3] Parsons JT, Horwitz AR, Schwartz MA. Cell adhesion: integrating cytoskeletal dynamics and cellular tension. Nat Rev Mol Cell Biol. 2010;11(9):633–43. doi: 10.1038/nrm2957 . 20729930 PMC2992881

[4] Choi CK, Vicente-Manzanares M, Zareno J, Whitmore LA, Mogilner A, Horwitz AR. Actin and alpha-actinin orchestrate the assembly and maturation of nascent adhesions in a myosin II motor-independent manner. Nat Cell Biol. 2008;10(9):1039–50. doi: 10.1038/ncb1763 . 19160484 PMC2827253

[5] Schoenwaelder SM, Burridge K. Bidirectional signaling between the cytoskeleton and integrins. Current Opinion in Cell Biology. 1999;11(2):274–86. doi: 10.1016/s0955-0674(99)80037-4 10209151

[6] Case LB, Waterman CM. Integration of actin dynamics and cell adhesion by a three-dimensional, mechanosensitive molecular clutch. Nat Cell Biol. 2015;17(8):955–63. Epub 20150629. doi: 10.1038/ncb3191 . 26121555 PMC6300998

[7] Nayal A, Webb DJ, Brown CM, Schaefer EM, Vicente-Manzanares M, Horwitz AR. Paxillin phosphorylation at Ser273 localizes a GIT1-PIX-PAK complex and regulates adhesion and protrusion dynamics. J Cell Biol. 2006;173(4):587–9. doi: 10.1083/jcb.200509075 . 16717130 PMC2063867

[8] Nordenfelt P, Elliott HL, Springer TA. Coordinated integrin activation by actin-dependent force during T-cell migration. Nat Commun. 2016;7:13119. Epub 20161010. doi: 10.1038/ncomms13119 . 27721490 PMC5062559

[9] Hughes PE, Pfaff M. Integrin affinity modulation. Trends Cell Biol. 1998;8(9):359–64. doi: 10.1016/s0962-8924(98)01339-7 . 9728397

[10] Calderwood DA, Shattil SJ, Ginsberg MH. Integrins and actin filaments: reciprocal regulation of cell adhesion and signaling. J Biol Chem. 2000;275(30):22607–10. doi: 10.1074/jbc.R900037199 . 10801899

[11] Giannone G, Dubin-Thaler BJ, Rossier O, Cai Y, Chaga O, Jiang G, et al. Lamellipodial actin mechanically links myosin activity with adhesion-site formation. Cell. 2007;128(3):561–75. doi: 10.1016/j.cell.2006.12.039 . 17289574 PMC5219974

[12] Zaidel-Bar R, Ballestrem C, Kam Z, Geiger B. Early molecular events in the assembly of matrix adhesions at the leading edge of migrating cells. Journal of Cell Science. 2003;116(22):4605–13. doi: 10.1242/jcs.00792 14576354

[13] Zhang X, Jiang G, Cai Y, Monkley SJ, Critchley DR, Sheetz MP. Talin depletion reveals independence of initial cell spreading from integrin activation and traction. Nat Cell Biol. 2008;10(9):1062–8. doi: 10.1038/ncb1765 . 19160486 PMC2746969

[14] Worth DC, Parsons M. Adhesion dynamics: mechanisms and measurements. Int J Biochem Cell Biol. 2008;40(11):2397–409. Epub 20080414. doi: 10.1016/j.biocel.2008.04.008 . 18485788

[15] Gupton SL, Waterman-Storer CM. Spatiotemporal feedback between actomyosin and focal-adhesion systems optimizes rapid cell migration. Cell. 2006;125(7):1361–74. doi: 10.1016/j.cell.2006.05.029 16814721

[16] Laukaitis CM, Webb DJ, Donais K, Horwitz AF. Differential dynamics of alpha 5 integrin, paxillin, and alpha-actinin during formation and disassembly of adhesions in migrating cells. J Cell Biol. 2001;153(7):1427–40. doi: 10.1083/jcb.153.7.1427 . 11425873 PMC2150721

[17] Zaidel-Bar R, Cohen M, Addadi L, Geiger B. Hierarchical assembly of cell-matrix adhesion complexes. Biochem Soc Trans. 2004;32(Pt3):416–20. doi: 10.1042/BST0320416 . 15157150

[18] Riveline D, Zamir E, Balaban NQ, Schwarz US, Ishizaki T, Narumiya S, et al. Focal contacts as mechanosensors: externally applied local mechanical force induces growth of focal contacts by an mDia1-dependent and ROCK-independent mechanism. J Cell Biol. 2001;153(6):1175–86. doi: 10.1083/jcb.153.6.1175 . 11402062 PMC2192034

[19] Katsumi A, Milanini J, Kiosses WB, del Pozo MA, Kaunas R, Chien S, et al. Effects of cell tension on the small GTPase Rac. J Cell Biol. 2002;158(1):153–64. Epub 20020708. doi: 10.1083/jcb.200201105 . 12105187 PMC2173027

[20] Galbraith CG, Yamada KM, Sheetz MP. The relationship between force and focal complex development. Journal of Cell Biology. 2002;159(4):695–705. doi: 10.1083/jcb.200204153 12446745 PMC2173098

[21] Puklin-Faucher E, Sheetz MP. The mechanical integrin cycle. J Cell Sci. 2009;122(Pt 2):179–86. doi: 10.1242/jcs.042127 . 19118210 PMC6518156

[22] Changede R, Xu XC, Margadant F, Sheetz MP. Nascent Integrin Adhesions Form on All Matrix Rigidities after Integrin Activation. Dev Cell. 2015;35(5). doi: 10.1016/j.devcel.2015.11.001 26625956

[23] Rossier O, Octeau V, Sibarita JB, Leduc C, Tessier B, Nair D, et al. Integrins beta1 and beta3 exhibit distinct dynamic nanoscale organizations inside focal adhesions. Nat Cell Biol. 2012;14(10):1057–67. Epub 20120930. doi: 10.1038/ncb2588 . 23023225

[24] Iwamoto DV, Calderwood DA. Regulation of integrin-mediated adhesions. Curr Opin Cell Biol. 2015;36:41–7. Epub 20150717. doi: 10.1016/j.ceb.2015.06.009 . 26189062 PMC4639423

[25] Pasapera AM, Schneider IC, Rericha E, Schlaepfer DD, Waterman CM. Myosin II activity regulates vinculin recruitment to focal adhesions through FAK-mediated paxillin phosphorylation. Journal of Cell Biology. 2010;188(6):877–90. doi: 10.1083/jcb.200906012 20308429 PMC2845065

[26] Wei WC, Lin HH, Shen MR, Tang MJ. Mechanosensing machinery for cells under low substratum rigidity. Am J Physiol Cell Physiol. 2008;295(6):C1579–89. Epub 20081015. doi: 10.1152/ajpcell.00223.2008 . 18923058

[27] Friedland JC, Lee MH, Boettiger D. Mechanically activated integrin switch controls alpha5beta1 function. Science. 2009;323(5914):642–4. doi: 10.1126/science.1168441 . 19179533

[28] Geiger B, Spatz JP, Bershadsky AD. Environmental sensing through focal adhesions. Nat Rev Mol Cell Bio. 2009;10(1):21–33. doi: 10.1038/nrm2593 19197329

[29] Oakes PW, Bidone TC, Beckham Y, Skeeters AV, Ramirez-San Juan GR, Winter SP, et al. Lamellipodium is a myosin-independent mechanosensor. Proc Natl Acad Sci U S A. 2018;115(11):2646–51. Epub 20180227. doi: 10.1073/pnas.1715869115 . 29487208 PMC5856528

[30] Stricker J, Beckham Y, Davidson MW, Gardel ML. Myosin II-Mediated Focal Adhesion Maturation Is Tension Insensitive. Plos One. 2013;8(7). doi: ARTN e70652 doi: 10.1371/journal.pone.0070652 23923013 PMC3726642

[31] Bershadsky AD, Ballestrem C, Carramusa L, Zilberman Y, Gilquin B, Khochbin S, et al. Assembly and mechanosensory function of focal adhesions: experiments and models. Eur J Cell Biol. 2006;85(3–4):165–73. Epub 20051219. doi: 10.1016/j.ejcb.2005.11.001 . 16360240

[32] Bidone TC, Skeeters AV, Oakes PW, Voth GA. Multiscale model of integrin adhesion assembly. PLoS Comput Biol. 2019;15(6):e1007077. Epub 20190604. doi: 10.1371/journal.pcbi.1007077 . 31163027 PMC6568411

[33] Bachir AI, Horwitz AR, Nelson WJ, Bianchini JM. Actin-Based Adhesion Modules Mediate Cell Interactions with the Extracellular Matrix and Neighboring Cells. Cold Spring Harb Perspect Biol. 2017;9(7). Epub 20170705. doi: 10.1101/cshperspect.a023234 . 28679638 PMC5495059

[34] Chrzanowska-Wodnicka M, Burridge K. Rho-stimulated contractility drives the formation of stress fibers and focal adhesions. J Cell Biol. 1996;133(6):1403–15. doi: 10.1083/jcb.133.6.1403 . 8682874 PMC2120895

[35] Geiger B, Bershadsky A. Assembly and mechanosensory function of focal contacts. Curr Opin Cell Biol. 2001;13(5):584–92. doi: 10.1016/s0955-0674(00)00255-6 . 11544027

[36] Han SJ, Oak Y, Groisman A, Danuser G. Traction microscopy to identify force modulation in subresolution adhesions. Nat Methods. 2015;12(7):653–6. Epub 20150601. doi: 10.1038/nmeth.3430 . 26030446 PMC4490115

[37] Arnold M, Cavalcanti-Adam EA, Glass R, Blummel J, Eck W, Kantlehner M, et al. Activation of integrin function by nanopatterned adhesive interfaces. Chemphyschem. 2004;5(3):383–8. doi: 10.1002/cphc.200301014 . 15067875

[38] Cavalcanti-Adam EA, Volberg T, Micoulet A, Kessler H, Geiger B, Spatz JP. Cell spreading and focal adhesion dynamics are regulated by spacing of integrin ligands. Biophys J. 2007;92(8):2964–74. Epub 20070202. doi: 10.1529/biophysj.106.089730 . 17277192 PMC1831685

[39] Popov K, Komianos J, Papoian GA. MEDYAN: Mechanochemical Simulations of Contraction and Polarity Alignment in Actomyosin Networks. PLoS Comput Biol. 2016;12(4):e1004877. Epub 20160427. doi: 10.1371/journal.pcbi.1004877 . 27120189 PMC4847874

[40] Oelz DB, Rubinstein BY, Mogilner A. A Combination of Actin Treadmilling and Cross-Linking Drives Contraction of Random Actomyosin Arrays. Biophys J. 2015;109(9):1818–29. doi: 10.1016/j.bpj.2015.09.013 . 26536259 PMC4643270

[41] Bidone TC, Jung W, Maruri D, Borau C, Kamm RD, Kim T. Morphological Transformation and Force Generation of Active Cytoskeletal Networks. PLoS Comput Biol. 2017;13(1):e1005277. Epub 20170123. doi: 10.1371/journal.pcbi.1005277 . 28114384 PMC5256887

[42] Vogel V, Sheetz M. Local force and geometry sensing regulate cell functions. Nat Rev Mol Cell Biol. 2006;7(4):265–75. doi: 10.1038/nrm1890 . 16607289

[43] Schwartz MA. Integrins and extracellular matrix in mechanotransduction. Cold Spring Harb Perspect Biol. 2010;2(12):a005066. Epub 20101117. doi: 10.1101/cshperspect.a005066 . 21084386 PMC2982167

[44] Geiger B, Yamada KM. Molecular architecture and function of matrix adhesions. Cold Spring Harb Perspect Biol. 2011;3(5). Epub 20110501. doi: 10.1101/cshperspect.a005033 . 21441590 PMC3101841

[45] Oakes PW, Beckham Y, Stricker J, Gardel ML. Tension is required but not sufficient for focal adhesion maturation without a stress fiber template. Journal of Cell Biology. 2012;196(3):363–74. doi: 10.1083/jcb.201107042 22291038 PMC3275371

[46] Bangasser BL, Shamsan GA, Chan CE, Opoku KN, Tuzel E, Schlichtmann BW, et al. Shifting the optimal stiffness for cell migration. Nat Commun. 2017;8:15313. Epub 20170522. doi: 10.1038/ncomms15313 . 28530245 PMC5458120

[47] Bangasser BL, Rosenfeld SS, Odde DJ. Determinants of maximal force transmission in a motor-clutch model of cell traction in a compliant microenvironment. Biophys J. 2013;105(3):581–92. doi: 10.1016/j.bpj.2013.06.027 . 23931306 PMC3736748

[48] Isomursu A, Park KY, Hou J, Cheng B, Mathieu M, Shamsan GA, et al. Directed cell migration towards softer environments. Nat Mater. 2022;21(9):1081–90. Epub 20220711. doi: 10.1038/s41563-022-01294-2 . 35817964 PMC10712428

[49] Motahari F, Carlsson AE. Pulling-force generation by ensembles of polymerizing actin filaments. Phys Biol. 2019;17(1):016005. Epub 20191213. doi: 10.1088/1478-3975/ab59bd . 31747656 PMC7190088

[50] Bendix PM, Koenderink GH, Cuvelier D, Dogic Z, Koeleman BN, Brieher WM, et al. A quantitative analysis of contractility in active cytoskeletal protein networks. Biophys J. 2008;94(8):3126–36. Epub 20080111. doi: 10.1529/biophysj.107.117960 . 18192374 PMC2275689

[51] Bangasser BL, Odde DJ. Master equation-based analysis of a motor-clutch model for cell traction force. Cell Mol Bioeng. 2013;6(4):449–59. doi: 10.1007/s12195-013-0296-5 . 24465279 PMC3896613

[52] Kumar A, Ouyang M, Van den Dries K, McGhee EJ, Tanaka K, Anderson MD, et al. Correction: Talin tension sensor reveals novel features of focal adhesion force transmission and mechanosensitivity. J Cell Biol. 2016;214(2):231. doi: 10.1083/jcb.20151001207062016c . 27432899 PMC4949446

[53] Wang X, Ha T. Defining single molecular forces required to activate integrin and notch signaling. Science. 2013;340(6135):991–4. doi: 10.1126/science.1231041 . 23704575 PMC3710701

[54] Zhang Y, Ge C, Zhu C, Salaita K. DNA-based digital tension probes reveal integrin forces during early cell adhesion. Nat Commun. 2014;5:5167. Epub 20141024. doi: 10.1038/ncomms6167 . 25342432 PMC4209443

[55] Kong F, Garcia AJ, Mould AP, Humphries MJ, Zhu C. Demonstration of catch bonds between an integrin and its ligand. J Cell Biol. 2009;185(7):1275–84. doi: 10.1083/jcb.200810002 . 19564406 PMC2712956

[56] Choquet D, Felsenfeld DP, Sheetz MP. Extracellular matrix rigidity causes strengthening of integrin-cytoskeleton linkages. Cell. 1997;88(1):39–48. doi: 10.1016/s0092-8674(00)81856-5 . 9019403

[57] Galbraith CG, Yamada KM, Galbraith JA. Polymerizing actin fibers position integrins primed to probe for adhesion sites. Science. 2007;315(5814):992–5. doi: 10.1126/science.1137904 . 17303755

[58] Thievessen I, Thompson PM, Berlemont S, Plevock KM, Plotnikov SV, Zemljic-Harpf A, et al. Vinculin-actin interaction couples actin retrograde flow to focal adhesions, but is dispensable for focal adhesion growth. J Cell Biol. 2013;202(1):163–77. doi: 10.1083/jcb.201303129 . 23836933 PMC3704983

[59] Swaminathan V, Fischer RS, Waterman CM. The FAK-Arp2/3 interaction promotes leading edge advance and haptosensing by coupling nascent adhesions to lamellipodia actin. Mol Biol Cell. 2016;27(7):1085–100. Epub 20160203. doi: 10.1091/mbc.E15-08-0590 . 26842895 PMC4814217

[60] Serrels B, Serrels A, Brunton VG, Holt M, McLean GW, Gray CH, et al. Focal adhesion kinase controls actin assembly via a FERM-mediated interaction with the Arp2/3 complex. Nat Cell Biol. 2007;9(9):1046–56. Epub 20070826. doi: 10.1038/ncb1626 . 17721515

[61] Calderwood DA, Zent R, Grant R, Rees DJ, Hynes RO, Ginsberg MH. The Talin head domain binds to integrin beta subunit cytoplasmic tails and regulates integrin activation. J Biol Chem. 1999;274(40):28071–4. doi: 10.1074/jbc.274.40.28071 . 10497155

[62] Vicente-Manzanares M, Zareno J, Whitmore L, Choi CK, Horwitz AF. Regulation of protrusion, adhesion dynamics, and polarity by myosins IIA and IIB in migrating cells. J Cell Biol. 2007;176(5):573–80. Epub 20070220. doi: 10.1083/jcb.200612043 . 17312025 PMC2064016

[63] Wehrle-Haller B. Assembly and disassembly of cell matrix adhesions. Curr Opin Cell Biol. 2012;24(5):569–81. Epub 20120719. doi: 10.1016/j.ceb.2012.06.010 . 22819514

[64] Ma LN, Li XT, Liu C. From generalized Langevin equations to Brownian dynamics and embedded Brownian dynamics. Journal of Chemical Physics. 2016;145(11). doi: Artn 114102 doi: 10.1063/1.4962419

[65] Gardel ML, Sabass B, Ji L, Danuser G, Schwarz US, Waterman CM. Traction stress in focal adhesions correlates biphasically with actin retrograde flow speed. J Cell Biol. 2008;183(6):999–1005. doi: 10.1083/jcb.200810060 . 19075110 PMC2600750

[66] Xu XP, Kim E, Swift M, Smith JW, Volkmann N, Hanein D. Three-Dimensional Structures of Full-Length, Membrane-Embedded Human alpha(IIb)beta(3) Integrin Complexes. Biophys J. 2016;110(4):798–809. doi: 10.1016/j.bpj.2016.01.016 . 26910421 PMC4776043

[67] Bell GI. Models for the specific adhesion of cells to cells. Science. 1978;200(4342):618–27. doi: 10.1126/science.347575 . 347575

[68] Pereverzev YV, Prezhdo OV, Forero M, Sokurenko EV, Thomas WE. The two-pathway model for the catch-slip transition in biological adhesion. Biophys J. 2005;89(3):1446–54. Epub 20050610. doi: 10.1529/biophysj.105.062158 . 15951391 PMC1366651

[69] Pereverzev YV, Prezhdo OV, Thomas WE, Sokurenko EV. Distinctive features of the biological catch bond in the jump-ramp force regime predicted by the two-pathway model. Phys Rev E Stat Nonlin Soft Matter Phys. 2005;72(1 Pt 1):010903. Epub 20050719. doi: 10.1103/PhysRevE.72.010903 . 16089930

[70] Lu F, Zhu L, Bromberger T, Yang J, Yang Q, Liu J, et al. Mechanism of integrin activation by talin and its cooperation with kindlin. Nat Commun. 2022;13(1):2362. Epub 20220429. doi: 10.1038/s41467-022-30117-w . 35488005 PMC9054839

[71] Schmidt CE, Chen T, Lauffenburger DA. Simulation of integrin-cytoskeletal interactions in migrating fibroblasts. Biophys J. 1994;67(1):461–74. doi: 10.1016/S0006-3495(94)80502-8 . 7522599 PMC1225379

[72] Kucik DF, Kuo SC, Elson EL, Sheetz MP. Preferential attachment of membrane glycoproteins to the cytoskeleton at the leading edge of lamella. J Cell Biol. 1991;114(5):1029–36. doi: 10.1083/jcb.114.5.1029 . 1874785 PMC2289124

