# Supplementary figures and images for: Computational model of integrin adhesion elongation under an actin fiber
Source: PLoS Comput Biol. 2023 Jul 6;19(7):e1011237. doi: 10.1371/journal.pcbi.1011237 (PMC10325090; doi:10.1371/journal.pcbi.1011237)

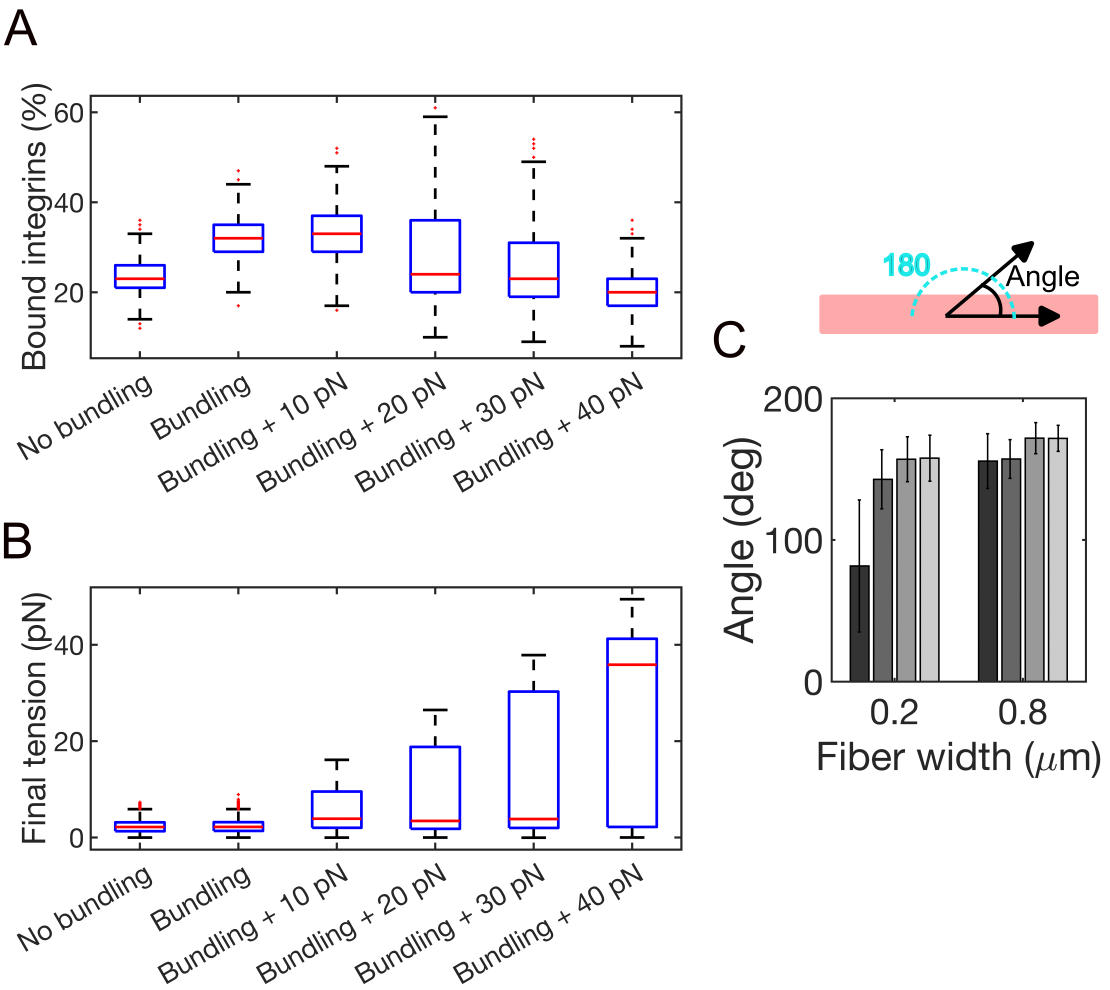

Supplement: S1 Fig — A. Distribution of the percentage of ligated integrins in different conditions of bundling and Fmyo (between 0–30 pN). B. Distribution of final tension on ligated integrins in different conditions of bundling and Fmyo (between 0–30 pN). All data are computed between 100–500 s of simulations, extracting values every 1 s. C. Average angle of the adhesion using Fmyo = 10 pN (black), 20 pN (dark gray), 30 pN (medium gray), 40 pN (light gray) and two fiber widths: 0.2 and 0.8 nm. Errorbars indicate standard deviation from the mean. The angle is calculated from the direction of the first principal component considering the 2D positions of ligated integrins. All data are computed between 100–500 s of simulations, using k = 0.6 pN/nm, and extracting values every 1 s. (TIFF) [file pcbi.1011237.s001.tiff]

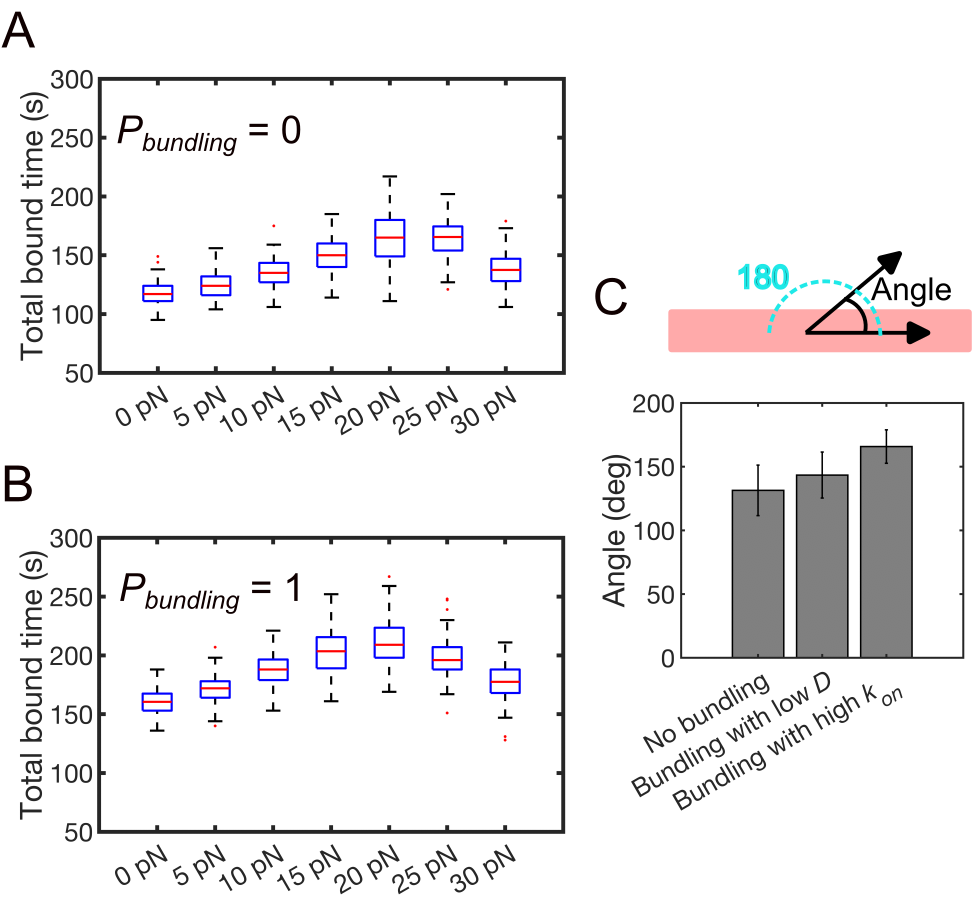

Supplement: S2 Fig — A-B. Distribution of the total time spent by integrins in the ligated state, in different conditions of Fmyo (between 0–30 pN), without (A) and with (B) actin bundling. C. Average angle of the adhesion relative to the actin fiber. Three conditions are tested: absence of bundling; bundling with reduced diffusion of integrin in the fiber region (using 5-fold higher ζi than outside the fiber); and bundling with increased activation rate (using 3-fold higher kon relative to kon outside the fiber). The angle is calculated from the direction of the first principal component considering the 2D positions of ligated integrins. All data are computed between 100–500 s of simulations, using k = 0.6 pN/nm, and recording every 1 s. (TIFF) [file pcbi.1011237.s002.tiff]

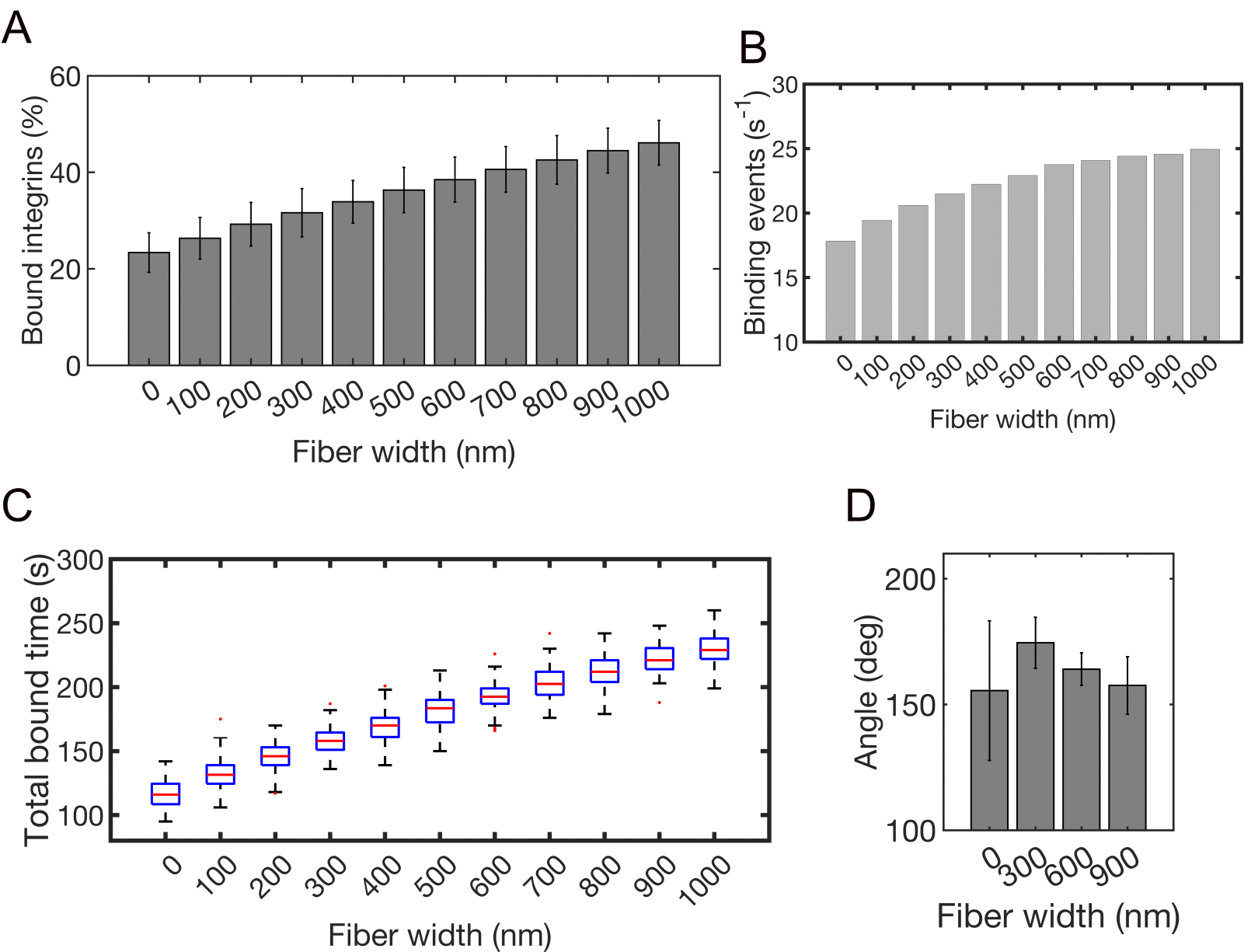

Supplement: S3 Fig — A. Average percentage of ligated integrins varying the width of the actin fiber and using Pbundling = 1. Errorbars indicate standard deviation from the mean. B. Number of binding events per second varying the width of the actin fiber and using Pbundling = 1. C. Distribution of total time in the ligated state, calculated as sum of the ligand-bound lifetimes of for each integrin over the course of 500 s of simulations, using Pbundling = 1 and increasing the width of the fiber. D. Average angle of integrin adhesion, relative to the direction of the actin fiber, using Pbundling = 1 and varying the width of the fiber. The angle is calculated from the direction of the first principal component of the 2D positions of ligated integrins. Errorbars indicate standard deviation from the mean. All data are computed in the absence of actomyosin contractility and are evaluated between 100–500 s of simulations, from 3 independent runs using k = 0.6 pN/nm and considering the total number of ligated integrins every 1 s of simulations. (TIFF) [file pcbi.1011237.s003.tiff]

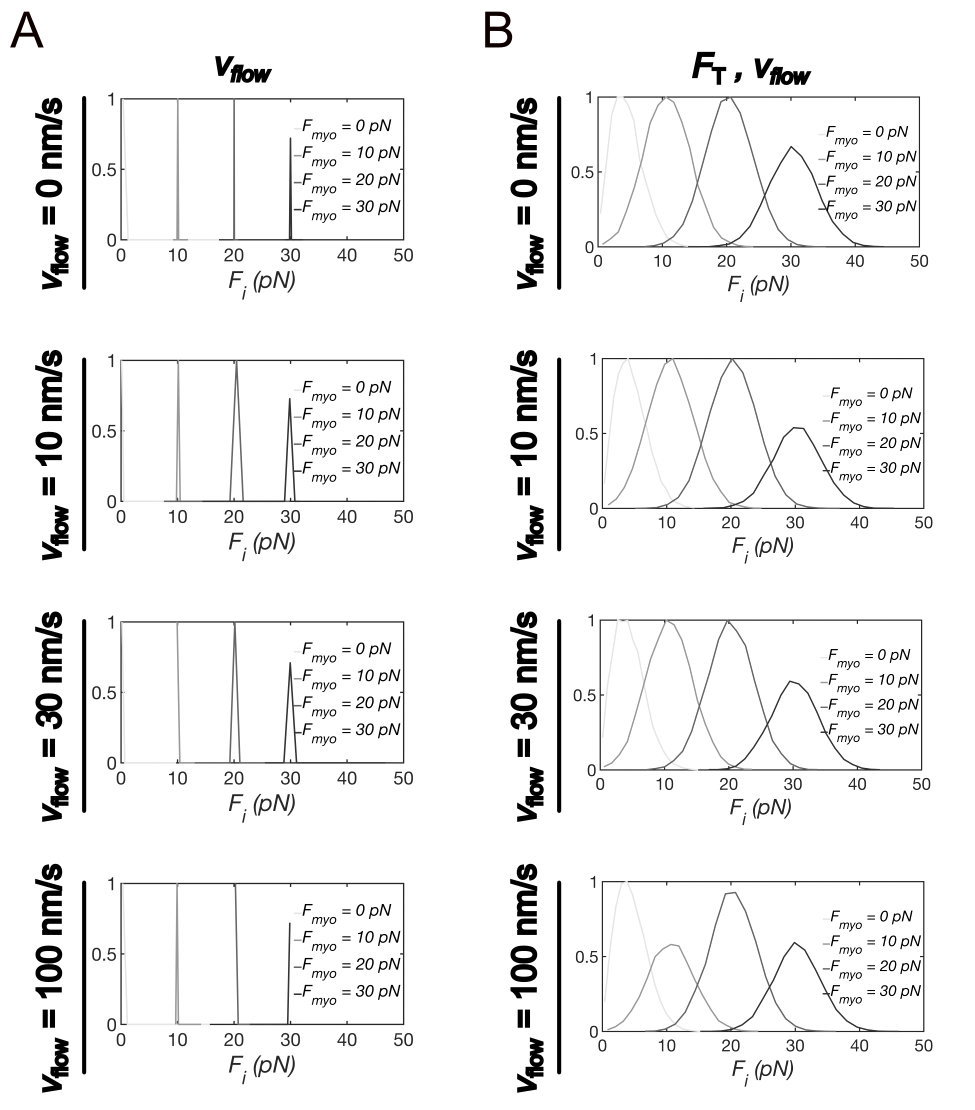

Supplement: S4 Fig — A. Distribution of force on ligated integrins using actin flow velocity of 0, 10, 30, and 100 nm/s, in different conditions of Fmyo (between 0–30 pN) and using FT = 0. B. Distribution of force on ligated integrins using actin flow velocity of 0, 10, 30, and 100 nm/s, in different conditions of Fmyo (between 0–30 pN) and including thermal fluctuations. All data are computed between 100–500 s, from 3 independent runs using k = 0.6 pN/nm. The distributions are normalized by their maximum value in each plot. (TIFF) [file pcbi.1011237.s004.tiff]

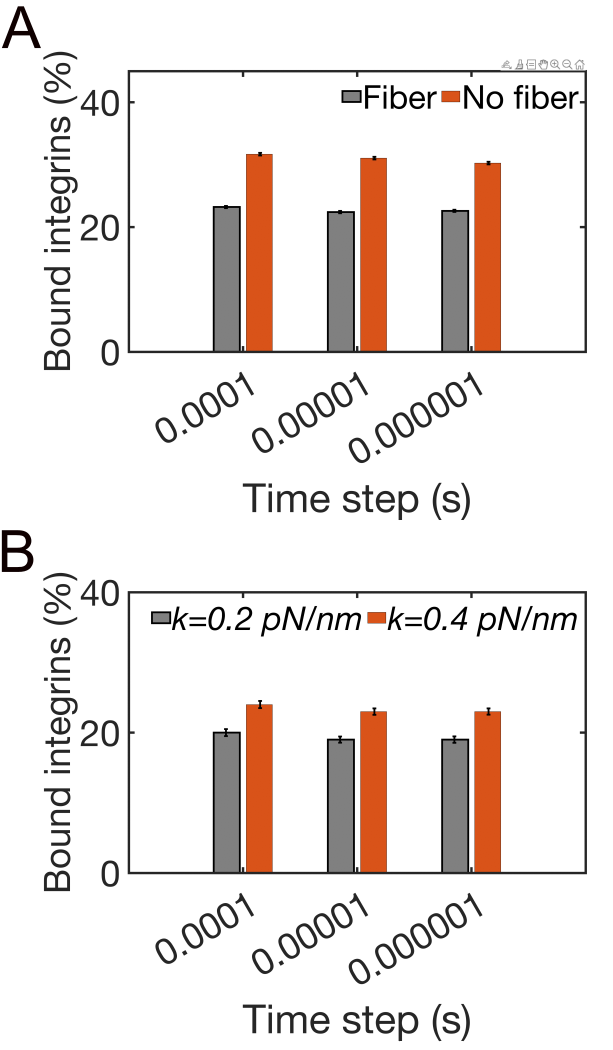

Supplement: S5 Fig — A. Average percentage of ligated integrins varying timestep, in the presence (fiber) or absence (no fiber) of actin bundling. When present, a fiber of 300 nm width is used. B. Average percentage of ligated integrins varying timestep, and using k = 0.2 pN/nm and k = 0.4 pN/nm. An actomyosin force of 20 pN is applied to ligated integrins in the fiber area, considering a fiber width of 300 nm. All data are computed between 100–500 s, from 3 independent runs. Errorbars indicate standard error from the mean. (TIFF) [file pcbi.1011237.s005.tiff]
